# Obstructive sleep apnea as a risk factor for primary open angle glaucoma and ocular hypertension in a monocentric pilot study

**DOI:** 10.1186/s12931-020-01533-7

**Published:** 2020-10-08

**Authors:** Katharina Bahr, Michael Bopp, Waeel Kewader, Henri Dootz, Julia Döge, Tilman Huppertz, Perikles Simon, Verena Prokosch-Willing, Christoph Matthias, Haralampos Gouveris

**Affiliations:** 1 Sleep Medicine Center, Department of Otorhinolaryngology, Head and Neck Surgery, Medical Center of the University of Mainz, 55131 Mainz, Germany; 2 Department of Ophthalmology, Medical Center of the University of Mainz, Mainz, Germany; 3 Institute for Sports Science, Johannes Gutenberg-University, Mainz, Germany

**Keywords:** Obstructive sleep apnea, Sleep-disordered breathing, Glaucoma, Primary open angle glaucoma

## Abstract

**Background:**

Both glaucoma and obstructive sleep apnea (OSA) are widespread diseases. OSA may presumably partly cause or worsen glaucoma, although the etiopathogenesis is unclear. Here we analyze for the first time the possible association between different glaucoma phenotypes and OSA.

**Methods:**

110 patients (47 females, 63 males; median age 64.3 years, median BMI 26.62 kg/m^2^) with suspected glaucoma and without any prior diagnosis of OSA were prospectively studied by one-night home sleep apnea testing (HSAT), 101 of the patients were analyzed. HSAT parameters, like apnea hypopnea index (AHI) and oxygen desaturation index as well as opthalmological parameters like intraocular pressure (IOP) and mean defect depth (MD) were collected. Moreover, HSAT results were compared across four phenotypic groups: primary open angle glaucoma (POAG), low-tension-glaucoma (LTG), ocular hypertension (OH), and controls.

**Results:**

There was no strong correlation between IOP or MD and AHI. BMI, age and gender did not differ between groups. Significant differences between POAG and LTG were found for all HSAT parameters. The AHI showed the most prominent group difference (Wilcoxon-Kruskal-Wallis rank sum test was highly significant with chi^2^ = 22, df = 3 *p* < 0.0001) with severely lower event rates in the LTG (9.45/h) compared to POAG (22.7/h) and controls (21.9/h; *p* < 0.0001 and 0.02, respectively). Highly significant differences were found between the four groups regarding AHI (Chi^2^ = 22, df = 3, *p* < 0.0001) with significantly lower events per hour in the LTG compared to POAG (Hodges-Lehmann = − 13.8, 95% CI (− 18.6 – − 8.8; *p* < 0.0001) and to controls (Hodges-Lehmann = 12.1, 95% CI -19.9 – − 2.4; *p* < 0.02). Severe and moderate OSA was more prevalent in POAG (69.8%) and OH (33.3%) than in LTG (9%). The effect of the glaucoma phenotype on the AHI was more prominent in females (*p* = 0.0006) than in males (*p* = 0.011).

**Conclusion:**

Although physical endpoints, such as MD and IOP, do not correlate with AHI, there was a strong correlation between the POAG and OH clinical glaucoma phenotypes and the AHI. Further studies should investigate the necessity to test routine screening for OSA by HSAT in patients with diagnosed POAG and OH. Besides, some characteristics of LTG differed widely from other glaucoma types and controls. LTG patients had a significantly lower rate of OSA compared to other glaucoma types and even controls. This might be due to a different pathogenesis of LTG.

**Trial registration:**

Retrospectively registered at DRKS (nr. S00021201) on April 9th 2020.

## Introduction/background

Obstructive sleep apnea (OSA) is a sleep disorder characterized by pauses in breathing or instances of shallow breathing during sleep (international classification of diseases –ICD- 10 code: G47.31). Glaucoma is a group of diseases characterized by damage to the optic nerve and retinal nerve fibers (ICD-10 code: H40-H42).

Both glaucoma and OSA are widespread diseases. Due to its current prevalence of 23% in women and 50% in men, public awareness of OSA steadily increases [1]. The disease is characterized by breathing interruptions, due to a narrowing of the upper respiratory tract causing reduced peripheral oxygen supply during sleep. Termination of these events requires arousal from sleep in order to re-establish the upper airway tone and resume ventilation, resulting in sleep fragmentation and thus poor sleep quality [2]. So far, a number of risk factors for OSA have been identified, such as obesity, increasing age and the associated reduced muscle tone, as well as anatomical compromise promoting upper airway narrowing [3].

Snoring, sleep disruption, impaired daytime performance, attention deficits and daytime sleepiness are characteristic symptoms of OSA. However, since in principle all organs can be affected by the nightly intermittent oxygen deficiency, further effects of the disease are also possible.

Glaucoma, on the other hand, is a group of eye diseases, in which damage to the optic nerve leads to visual field disturbances. This damage is caused by a multifactorial progressive optic neuropathy that can result in the degeneration of retinal ganglion cells, leading to a characteristic cupping of the optic nerve and thus in irreversible vision loss and blindness [4]. A distinction is made between different glaucoma phenotypes, whereby a major factor is intraocular pressure. Increased intraocular pressure is considered the only modifiable risk factor, as opposed to other non-modifiable risk factors, like age and positive family history [5]. However, not every glaucoma phenotype is associated with high intraocular pressure; e.g. in the low-tension glaucoma (LTG) phenotype damage to the optic nerve occurs without increased intraocular pressure (IOP).

A link between OSA and glaucoma has been proposed [5–10]. Specifically, it is assumed that OSA might partly cause or worsen glaucoma [5]. To date, the exact pathogenesis is unclear. One suggested mechanism is episodic hypoxic damage of the optic nerve [11]. Chaitanya et al. have reviewed current theories and have distinguished between hypoxia and vascular and/or mechanical factors [12]. Intermittent hypoxia caused by OSA leads to optic nerve damage due to oxygen undersupply. According to the authors [12], vascular pathological changes that are demonstrably caused by OSA also lead to optic nerve neurodegeneration due to nerve hypoperfusion. A further mechanistic connection is caused by an increased intraocular pressure due to the mechanical breathing instability associated with OSA. Another hypothesis linking OSA and glaucoma involves increased intermittent sympathetic tone during sleep which is due to the respiratory events and associated arousals, that leads to an increased release of vasoconstrictive substances (catecholamines, angiotensin II, vasopressin) and atrial natriuretic peptide (ANP) [13]. However, the extent of the role played by OSA in the development of glaucoma is still rather unclear.

Although there are several studies correlating the severity of glaucoma with the severity of OSA, there are only few published investigations of the possible correlations between different glaucoma phenotypes and OSA severity [14]. The aim of the present investigation is to study the correlation of the severities of both entities, e.g. establishing a correlation of ophthalmological parameters, namely IOP as one of the main risk factors and the mean defect (MD) in visual fields, as a marker for severity of the disease, with quantifiable parameters of OSA like the apnea/hypopnea index (AHI) and the oxygen desaturation index (ODI). Besides that, distinct glaucoma phenotypes were analyzed regarding the respective OSA prevalence. Moreover, we discuss the potential interactions and the pathogenesis of OSA and glaucoma and make suggestions for their clinical management.

## Methods

**Fig. 1:**
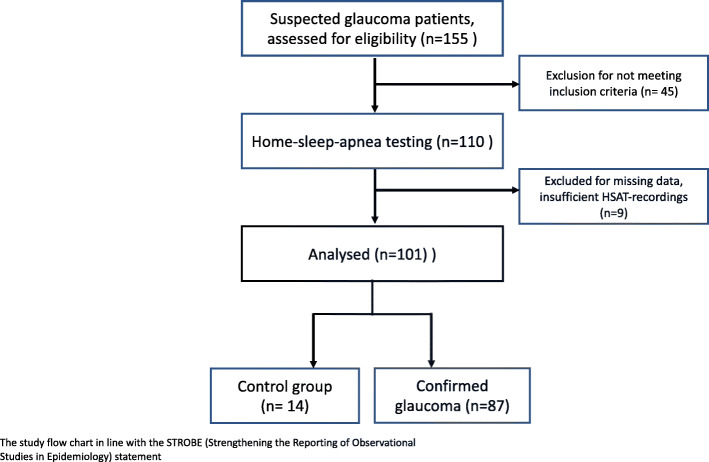
The study flow chart in line with the STROBE (Strengthening the Reporting of Observational Studies in Epidemiology) statement

In order to correlate ophthalmological parameters (in particular MD and IOP) with OSA-related respiratory home sleep apnea testing (HSAT) parameters, glaucoma patients and patients with ocular hypertension (OH) with no previous diagnosis of OSA who were hospitalized for glaucoma diagnosis were prospectively recruited to undergo an attended one-night-HSAT screening for OSA. Glaucoma-specific diagnostic procedures included a full ophthalmological examination, repeated IOP measurements during different specified times of the day in a period of 36 h and MD measurements via perimetry. Inclusion criteria for this prospective study were a diagnosis of glaucoma or ocular hypertension made by a dedicated glaucoma consultant, positive snoring history, age > 18 years, patient’s compliance and insight and body mass index (BMI) ≤34.9 kg/m^2^. Exclusion criteria were active malignant tumors (end of treatment < 5 years), age < 18 years, COPD (Stadium Gold 2–4) Raynaud’s phenomenon (causing problems of pulse oxymetry measurements) and BMI > 34.9 kg/m^2^. Participants who were initially suspected to suffer from glaucoma or ocular hypertension but eventually did not meet the criteria either for glaucoma or for ocular hypertension formed the control group. The process of recruitment and analysis is shown as a STROBE flowchart in Fig. 1.

Relevant parameters in HSAT (polygraphy) were apnea-hypopnea-index (AHI), oxygen desaturation index (ODI) and average and minimal arterial oxygen desaturation as measured with pulse oxymetry. Ophthalmological parameters were intraocular pressure (IOP) in mmHg for each eye and mean defect depth (MD) as well as the glaucoma phenotype, namely primary open angle glaucoma (POAG), low-tension-glaucoma (LTG) or preliminary stages of high-tension glaucoma - so-called ocular hypertension (OH). General parameters included in the analysis were age, gender and BMI.

Miniscreen plus (Loewenstein Medical, EC-certificate acc.93/42/ECC Annex II) HSAT devices were used, equipped with a pulse oxymeter, nasal cannula and piezoelectric straps for recording of chest and abdomen excursion. Before falling asleep, study participants put on the device themselves after being instructed a few hours earlier. This device technology provides a 70–85% validity and efficiency in detecting OSA [15], and has a tendency to overestimate the sleep period by 20% and underestimate OSA severity by 11% [16]. Albeit the mentioned shortcomings when using this type of HSAT devices, we decided to use HSAT technology –and not polysomnography- to diagnose OSA. This was because of its lower cost, its greater convenience for the patients and due to the fact, that it is an established and approved method for the diagnosis of OSA and consequential treatment decisions.

Participants entered the study after giving consent and being informed about the purpose of the study. The study protocol and the procedures used were in accordance with the principles of the Declaration of Helsinki. The study was approved by the local ethics committee of the respective state medical association (Number 837.068.17 [10902]).

### Statistical analysis

Group comparisons were done by Kruskall-Wallis testing. Non-parametric comparisons between groups as post-hoc testing were done using the Wilcoxon’s test and Hodges-Lehmann values including 95%-confidence intervals. Bonferroni corrected *p*-values were also calculated. Wilcoxon’s/Kruskal- Wallis rank sum test was performed to compare the different glaucoma phenotypes in terms of OSA-parameters, like AHI, apnea-index (AI), hypopnea-index (HI) and oxygen desaturation index (ODI) as shown on Table 4. Scaling of OSA severity between mild (AHI 5–15/h), moderate (AHI ≥15–30/h), severe (AHI ≥ 30/h) and non-OSA (AHI ≤ 5 /h) was done. We compared their prevalence in the different glaucoma phenotypes with contingency analyses.

## Results

A total of 110 glaucoma patients (47 females, 63 males; median age 64.3 years, median BMI 26.62 kg/m^2^) have been screened via HSAT. After screening, 101 individuals were found to meet the inclusion criteria for analysis. Table 1 shows the demographic features of the analyzed cohort. Eventually, 14 participants who were initially suspected to suffer from glaucoma or ocular hypertension but did not meet the criteria either for glaucoma or for ocular hypertension comprised the control group.

**Table 1:** Epidemiologic features and ranges of AHI, and ODI of the analyzed groups

Group	Gender (f/m)	Age median (Quartiles)	BMI median (Quartiles)	AHI median (Quartiles)	ODI median (Quartiles)
POAG	21/31	67 (55–77)	26 (24–29)	22.7 (12.4–30.3)	21.8 (12.2–29.0)
LTG	14/8	63.5 (57.8–73.5)	25 (24–27)	9.45 (5.7–11.4)	10.6 (8.1–17.3)
OH	4/10	64.5 (50.5–68)	29 (24–31)	10.9 (5.9–29.1)	14.65 (6.7–19.8)
Control	8/5	70 (56–73)	26 (24–31)	21.9 (6.1–30.5)	16.7 (7.7–29.5)

Due to the quite different variance of the respective values within each one of the groups, median values and quartiles are presented. *POAG* Primary open angle glaucoma, *LTG* Low-tension glaucoma, *OH* Ocular hypertension, *AHI* Apnea hypopnea index, *ODI* Oxygen desaturation index, *BMI* Body mass index.

There was no significant correlation between both intraocular pressure (IOP) or median defect depth (MD) and the AHI. BMI and age did not influence the results. Different glaucoma phenotypes, namely primary open angle glaucoma (POAG), low-tension-glaucoma (LTG) and ocular hypertension (OH), were identified and analyzed separately in terms of AHI and OSA prevalence (shown on Tables 2 and 3). Since the data related sleep apnea showed signs of non-normal distribution and unequal variance across groups, we used Wilcoxon-Testing providing respective Z-values as well as Hodges-Lehmann estimator as a rank based estimator for the median difference between two groups “Group” subtracted by “-Group” (Tables 3, 4 and 5).

**Table 2:** Contingency Table: Distribution of obstructive sleep apnea (OSA) severity levels by glaucoma group; AHI = apnea-hypopnea-index

	Mild AHI 5–15/h	Moderate AHI ≥15–30/h	Severe AHI ≥ 30/h	No OSA AHI ≤ 5 /h	Total (%)
Primary open angle glaucoma	11	23	14	5	53 (52.48)
Low-tension glaucoma	16	2	0	4	22 (21.78)
Ocular hypertension	6	2	2	2	12 (11.88)
No Glaucoma	2	6	3	3	14 13.86)
**Total (%)**	**35 (34.65)**	**33 (32.67)**	**19 (18.81)**	**14 (13.86)**	**101 (100)**

**Table 3:** Non-parametric comparisons with Wilcoxon-Method

Analyzed Parameter	Group	- Group	Z	p-value	Hodges-Lehmann	Chi-Square-Approximation: Probability > Chi^**2**^
AHI	Control	LTG	2.4	0.019	12.1	< 0.0001
AHI	LTG	POAG	−4.9	< 0.001	−13.8	
AI	Control	LTG	2.2	0.0253	4.1	0.0054
AI	LTG	POAG	−3.50	0,0004	−6.5	
HI	Control	LTG	2.4	0.0176	6.6	0.0024
HI	OH	LTG	2.3	0.0203	2.9	
HI	LTG	POAG	−3.6	0.0003	−4.9	
SI	LTG	POAG	−2.4	0.0183	−3.9	0.0741
ODI	OH	POAG	−2.3	0.0207	−7.4	0.0025
ODI	LTG	POAG	−3.6	0.0004	−9.2	

*AHI* Apnea hypopnea index, *AI* Apnea index, *HI* Hypopnea index, *SI* Snoring index, *ODI* Oxygen Desaturation index, *LTG* Low-tension-glaucoma, *POAG* Primary open-angle-glaucoma, *OH* Ocular hypertension. Positive Hodges-Lehmann values represent positive differences between “Group” and “-Group”.

**Table 4:** Non-parametric comparisons with Wilcoxon-Method in females

Analyzed Parameter	Group	- Group	Z	***p***-value	Hodges-Lehmann	Chi-Square-Approximation: Probability > Chi^**2**^
AHI	Controls	POAG	−2.3	0.0233	−11	−0.0006
AHI	LTG	POAG	−4.1	< 0.0001	−14.8	
AI	LTG	POAG	−3.0	0.0032	−6.5	0.0195
HI	LTG	POAG	−2.9	0.0032	−6.9	0.0199
ODI	Control	POAG	−2.2	0.0281	−9.0	0.0183
ODI	LTG	POAG	−2.7	0.0078	−8.9	

*AHI* Apnea hypopnea Index, *AI* Apnea Index, *HI* Hypopnea Index, *ODI* Oxygen Desaturation Index, *LTG* Low-tension-glaucoma, *POAG* Primary open-angle-glaucoma. Positive Hodges-Lehmann values represent positive differences between “Group” and “-Group”.

**Table 5:** Non-parametric comparisons with Wilcoxon-Method in males

Analyzed Parameter	Group	- Group	Z	***p***-value	Hodges-Lehmann	Chi-Square-Approximation: Probability > Chi^**2**^
AHI	Control	LTG	2.9	0.0042	22.6	0.0111
AHI	LTG	POAG	−2.5	0.0117	−12.4	
AI	Control	LTG	2.3	0.0233	12.9	0.0958
HI	Control	POAG	2.2	0.0316	8.6	0.0141
HI	Control	LTG	2.9	0.0043	11.7	
HI	Control	OH	2.3	0.0235	9.6	
ODI	Control	LTG	2.6	0.0103	9.7	0.015
ODI	Control	OH	2.0	0.0433	15.7	
ODI	LTG	POAG	−2.3	0.0198	−10.0	

*AHI* Apnea hypopnea Index, *AI* Apnea Index, *HI* Hypopnea Index, *SI* Snoring Index, *ODI* Oxygen Desaturation Index, *LTG* Low-tension-glaucoma, *POAG* Primary open-angle-glaucoma, *OH* Ocular hypertension. Positive Hodges-Lehmann values represent positive differences between “Group” and “-Group”.

**Fig. 2:**
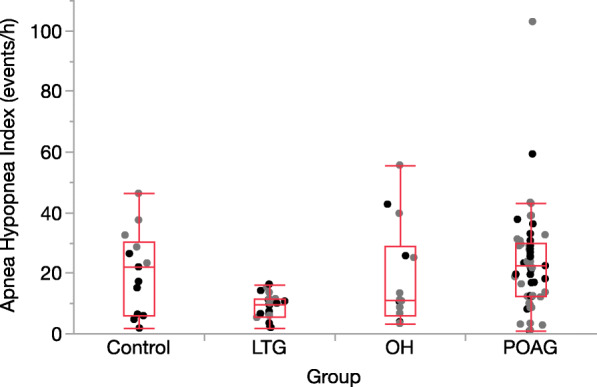
Distribution of the apnea hypopnea index in different glaucoma groups; females in black, males in grey. OSA severity was classified as mild (AHI 5–15/h), moderate (AHI ≥15–30/h), severe (AHI ≥ 30/h) and non-OSA (AHI ≤ 5 /h). POAG = primary open angle glaucoma, LTG = low-tension glaucoma, OH = ocular hypertension

The presented *p*-value is the post-hoc *p*-Value for the comparison of the groups, while the “ Probability >Chi^2^” provides the p-value for the global group comparison across the four different groups (control, TLG, POAG, OH) for the respective “analyzed parameter”. Obstructive sleep apnea was significantly more prevalent in patients with high IOP, namely participants with POAG or OH, whereas participants with LTG had significantly fewer and less severe OSA cases. The median AHI was 22.7/h in the POAG group and 21.9/h in the in OH group. In contrast, the median AHI in LTG was 9.45/h (*p* = 0.0009) (Fig. 2). Highly significant differences were found between the four groups with regard to the AHI (Chi^2^ = 22, df = 3, *p* < 0.0001) with significantly fewer events per hour in the LTG compared to POAG (Hodges-Lehmann = − 13.8, 95% CI (− 18.6 – − 8.8; *p* < 0.0001) and the controls (Hodges-Lehmann = 12.1; 95% CI -19.9 – − 2.4; *p* < 0.02). Additionally, this effect was much more obvious in females (*p* = 0.0006) than in males (*p* = 0.011) (Fig. 2).

## Discussion

In this report evidence is provided that only glaucoma phenotypes that are associated with increased IOP show higher AHI. Obstructive sleep apnea was significantly more prevalent in patients with high IOP, namely participants with POAG or OH. Other glaucoma phenotypes, like LTG, that by definition also show a damage of the optic nerve but are not associated with increased IOP, are not associated with OSA. To our knowledge, this is the first report dedicated to the investigation of the association of OSA with different glaucoma phenotypes. Nonetheless, AHI did not correlate directly with physical measures associated with glaucoma, namely IOP or MD.

It may be suggested, that OSA, in particular moderate and severe OSA forms, leads to higher IOP and thus to the two aforementioned phenotypes. However, higher AHI did not correlate with higher IOP. The reason why glaucoma phenotypes correlate with OSA severity, instead of physical measures associated with glaucoma, such as IOP or MD, remains unclear. Notably, this finding is in line with the few studies that showed a lack of correlation of IOP with the AHI/RDI [17]. For example, Geyer et al. could neither find a higher incidence of OSA in open-angle glaucoma nor a correlation of the RDI with IOP. Xin et al. on the other hand showed that OSA had a significantly higher prevalence in POAG and in particular severe OSA was associated with higher IOP and optic nerve damage, although no correlation could be drawn [18].

Women present a higher degree of systemic inflammation than males despite the same degree of OSA gravity [19]. In this present study OSA was even more clearly associated with hypertensive glaucoma types in women than in men. This should be considered when women are diagnosed with glaucoma or ocular hypertension.

There are already various studies dealing with a possible correlation between the two diseases. In spite of some large-scale studies and meta-analyses, the facts have not yet been fully explored. In their publication Shi et al. (2015) prepared a meta-analysis from 16 studies with a total of 2,278,832 participants [8]. The pooled analysis showed a statistically relevant correlation between glaucoma and sleep apnea with an OR (odds ratio) of 1.96 in the case-control studies and an OR of 1.67 in the retrospective cohort study. There was no distinction between glaucoma types and it is not clear whether the patients were naïve to OSA therapy. OSA diagnosis was also not uniformly made via in-home polysomnography, but via questionnaires, HSAT (polygraphies) and / or polysomnography or even just with the question “do you have sleep apnea”.

In a multicenter study from France with 9580 participants, Aptel et al. (2014) could not identify any statistically relevant relationship between the two diseases (OR 1.13) [20]. However, not all ophthalmological parameters had been included in that study. There could have been unknown glaucoma cases included in their cohort. Besides, there was no differentiation between glaucoma phenotypes and there was no information on possible exclusion of patients with previous OSA treatment. Moreover, only patients aged over 50 years had been included in that study. Additionally, Morsy et al. (2019) found in their study with 80 participants that OSA is associated with a higher risk of vision-threatening and non-threatening ocular disorders. The lowest oxygen desaturation index in OSA patients was a significant predictor of vision-threatening disorders [9]. In our study, on the other hand, highest ODIs were found in the POAG group, which made it possible to distinguish between POAG and OH as well as POAG and LTG (Table 3). Our patients were naïve to OSA therapy and built an appropriate cohort for further follow-up after initiation of OSA-therapy. To the best of our knowledge, this is the first prospective study that investigated glaucoma patients for the absence or presence of OSA. Most cohort studies and meta-analyses analyzed OSA cohorts in terms of the presence of glaucoma. Glaucoma criteria were often not specified and there was often no distinction between the different glaucoma phenotypes.

Weaknesses of this study are the rather sparse background information on other concomitant, e.g. cardiovascular, diseases of the glaucoma patients and on previously performed glaucoma therapy (e.g. previous outpatient procedures), which were not documented in detail in the medical records and which could hence have been a confounder of the results. Moreover, a further limitation of the study is that full-night polysomnography was not performed. The sensors included in the HSAT device used in our study were also unable to detect hypopneas that are only associated with cortical arousals. Due to these limitations, such a HSAT device may underestimate the severity of OSA [21]. Notably, we did not use automatically scored HSAT data; on the contrary, the raw data from the HSAT device were reviewed and interpreted by physicians who are either board-certified in sleep medicine or overseen by a board-certified sleep medicine physician [22].

Further studies should include a detailed medical history of the patients and should include a follow-up of glaucoma- and OSA-specific parameters under OSA-therapy, e.g. positive-airway-pressure (PAP). Besides, different glaucoma therapies, surgical or pharmacological, should be additionally considered in the analysis and patients could be followed-up in terms of the evolution of their AHI (with or without PAP-therapy).

On the basis of these results, it can be suggested that glaucoma phenotypes with increased IOP and even ocular hypertension are associated with a higher AHI in snoring patients and thus with a higher risk of OSA. Moreover, it is suggested that OSA, and in particular its severe forms, may lead to an increase in IOP and thus to high IOP-associated clinical glaucoma phenotypes. There is also a remarkable negative association between low-tension-glaucoma and OSA, since there was not a single case of severe OSA in the group of LTG-participants. This fact may suggest that low-tension-glaucoma pathogenesis is quite different from the one involving high-tension glaucoma phenotypes. A further possibility appears to be that high-IOP glaucoma phenotypes and OSA may share common pathogenetic mechanisms or that IOP- and AHI- measures may be subject to the same confounders.

One such possible common pathogenetic mechanism may involve inflammatory pathways that are more relevant to the development of optic nerve neuropathy and loss of vision in POAG than in LTG. A higher level of systemic inflammation in OSA patients, typically associated with higher systemic blood pressure, may therefore only explain the pathogenesis of POAG. However, a large population-based Chinese cohort study could not find any association between the different glaucoma phenotypes and systemic inflammation, as depicted by C-reactive-protein values [23]. Other authors have speculated that an elevated neutrophil-to-lymphocyte ratio and the systemic immune inflammation index might serve as predictors for POAG [24]. Kondkar et al. found high systemic level of the inflammatory cytokine TNF-α in their study of 51 POAG patients, compared to 88 controls [25]. Nonetheless, the mechanisms linking increased intraocular pressure to increased systemic inflammation have not been sufficiently elucidated so far.

## Conclusion

Ophthalmologic parameters like intraocular pressure or mean defect depth were not strongly associated with the AHI, but there was a strong correlation between the clinical classification of the glaucoma phenotypes and the AHI. Especially the group of patients with LTG differed significantly from the group with POAG and healthy controls in terms of OSA-defining parameters, such as the AHI. We conclude that OSA leads to an increase of IOP and to high-IOP associated phenotypes, namely POAG and OH. Moreover, there is no correlation between OSA and LTG, which might support a different pathogenesis of LTG. We propose that further studies should be done to investigate the necessity for routine screening for OSA by HSAT specifically targeted to patients with definite POAG and definite ocular hypertension.

## Data Availability

All data generated or analysed during this study are included in this published article.

## Abbreviations

AHI: Apnea hypopnea index
AI: Apnea index
BMI: Body mass index
CRP: C-reaktive-protein
HI: Hypopnea index
IOP: Intra-ocular pressure
LTG: Low-tension glaucoma
MD: Mean defect depth
ODI: Oxygen desaturation index
OH: Ocular hypertension
OSA: Obstructive sleep apnea
PAP: Positive airway pressure
POAG: Primary open angle glaucoma
